# Significant difference in Th1/Th2 paradigm induced by tuberculosis-specific antigens between IGRA-positive and IGRA-negative patients

**DOI:** 10.3389/fimmu.2022.904308

**Published:** 2022-08-31

**Authors:** Qiang Li, Weicong Ren, Jinfeng Yuan, Haiping Guo, Yuanyuan Shang, Wei Wang, Junhua Pan, Mengqiu Gao, Yu Pang

**Affiliations:** ^1^ Department of Tuberculosis, Beijing Chest Hospital, Capital Medical University/Beijing Tuberculosis & Thoracic Tumor Research Institute, Beijing, China; ^2^ Department of Bacteriology and Immunology, Beijing Key Laboratory on Drug-Resistant Tuberculosis Research, Beijing Chest Hospital, Capital Medical University/Beijing Tuberculosis & Thoracic Tumor Research Institute, Beijing, China

**Keywords:** cytokine, interferon-γ release assays, tuberculosis, T cell, diagnosis

## Abstract

False negative interferon-γ release assay (IGRA) results constitute the major dilemma for the diagnosis of tuberculosis (TB) infections. Herein, we conducted a cohort study to compare the host immunological response to TB-specific antigens between active TB patients with positive and negative IGRA results and control groups. A total of 274 laboratory-confirmed TB patients were included in our analysis, consisting of 221 were IGRA positive and 53 were IGRA negative. Patients with the elderly were identified as an independent risk factor for negative IGRA results. In addition, the elevated level of IL-4 and the decreased levels of IFN-γ, IL-2, IL-6, IL-1β, and IL-12 in IGRA negative TB relative to IGRA positive TB group, demonstrating a significant difference in Th1/Th2 paradigm between two groups. The IFN-γ&IL-2 based assay could correctly identify 247 out of 307 MTB-infected individuals [271 TB patients and 36 individuals with latent TB infection (LTBI)], demonstrating a sensitivity of 80.5%. Then the IFN-γ and IL-4 were applied to distinguish healthy control and IGRA-negative group. When using the stepwise algorithm, the sensitivity for detecting *Mycobacterium tuberculosis* (MTB) infections was significantly increased from 80.5% to 89.6%. Additionally, patients with negative IGRA results had a conversion to culture-negative status longer than those with positive IGRA results. In conclusion, a stepwise algorithm outperforms IGRA assays to accurately identify MTB infections by the combination IFN-γ, IL-2, and IL-4. Further study is needed to evaluate the accuracy of our diagnostic algorithm in the LTBI population.

## Introduction

TB is a substantial global threat causing an estimated 10.0 million incident cases and 1.5 million deaths worldwide each year (1). It can be transmitted to susceptible individuals through sharing air space with individuals expelling aerosols with viable MTB bacilli (2). The vast majority of those infected are from developing countries, especially impoverished populations living at high density (3). To accelerate multisectoral response to TB control, the World Health Organization has declared the End TB Strategy, which aims at a 90% reduction in TB incidence rate by 2035 (4). A key strategy to achieve the elimination of TB is to improve the timely identification of active TB cases, and prevent the spread of the disease in the community (5).

The diagnosis of pulmonary TB relies on the bacteriological examination of sputum by smear microscopy and mycobacterial culture (6). Smears lack sensitivity and cannot differentiate live from dead bacilli (7). Mycobacterial culture yields high sensitivity; however, its prolonged turn-around time fails to meet clinician’s requirements (8). Recently, the commercial molecular test outperforms conventional bacteriological examination due to its high sensitivity and specificity apart from being rapid in diagnosis (6); but the molecular test cannot fully exclude active TB cases when the result is negative. A recent nationwide survey in China demonstrated that approximately two-thirds of active TB patients are without bacteriological evidence (9). Thus, host immune biomarkers and antigen detection from body fluid specimens will have a future role in diagnosis of these patients (10).

IGRAs are conducted as adjunctive tests in the diagnosis of active TB (11). Although these assays cannot discriminate between active TB diseases and LTBI, the negative IGRA results are considered promising criteria for exclusion of active TB based on their high negative predictive value (12). However, a systematic review and meta-analysis demonstrated that approximately one-tenth of individuals presenting with active TB have negative IGRA results (13). By retrospectively reviewing IGRA results in active TB patients, multiple factors were associated with negative IGRA results, including immunodeficiency, advanced age, and low lymphocyte count (13–15). However, the immunological mechanisms for these false-negative results remain unclear. The knowledge gaps in the field prompted us to explore immune response profiles to antigens of MTB in this population, which will provide new insights into the development of an immune biomarker for better diagnosis of active TB (10).

In response to MTB infection, a series of pro-inflammatory cytokines, such as TNF-α, IL-1β, IL-6, and IL-2 are upregulated *via* multiple immune signaling pathways (16). As these cytokines are synthesized and transported in human peripheral blood and are easy to be detected. Thus, we hypothesized that the detection of cytokines might correct for false-negative errors by IGRA assays and provide a new way to detect MTB infection. To test this hypothesis, we conducted a cohort study to compare the host immunological response to TB-specific antigens and the expression of multiple cytokines between active TB patients with negative IGRA results and control groups. The objective of this study was to determine the appropriate methods to correct for false-negative errors by IGRA assays and identify risk factors associated with false-negative IGRA results.

## Methods

### Patients

Between May 2020 and September 2021, we consecutively included laboratory-confirmed TB patients seeking health care in the Beijing Chest Hospital. Sputum and blood specimens from patients with symptoms suggestive of active TB were collected for smear microscopy, BACTEC MGIT 960 (Becton, Dickinson and Company, USA), GeneXpert MTB/RIF assay (Cepheid, Sunnyvale USA), and IFN-γ release assays (IGRAs, Deaou, Guangzhou China). The diagnosis of clinical TB patients was made on the basis of history, physical examination, and detection of tubercle bacilli in sputum using BACTEC MGIT 960 and GeneXpert MTB/RIF assay. Only the active TB patients with positive results that demonstrated the presence of MTB organisms in sputum were included in our cohort. Then, the TB patients were classified into IGRA-positive and IGRA-negative groups according to the IGRA results. After initiation of anti-TB treatment, sputum smears and BACTEC MGIT960 culture were recommended to be performed monthly during the first six-month period. The patients afflicted with RIF-resistant TB by laboratory evidence were excluded. In addition, we recruited healthy control and latent TB participants from the healthcare workers in Beijing Chest Hospital. The health-care workers who had abnormal chest radiography without any clinical symptoms suggestive of TB were defined as LTBI according to positive IGRA results. Healthy controls, without a known history of exposure to MTB, who had normal chest radiography without clinical features, were confirmed to have negative results on the IGRA test. Details of individuals enrolled are summarized in **Table 1**. This study was approved by the Ethics Committee of Beijing Chest Hospital, Capital Medical University.

**Table 1:** Demographic characteristics of participants included in the final cohort.

Parameters	HC	LTBI	TB IGRA(+)	TB IGRA (-)	Crude OR(95%CL)	p value	Adjusted OR(95%CL)	p value
	(n=39)	(n=36)	(n=221)	(n=53)				
Age, y	35.3 (18-54)	39.56 (26-53)	46.3 (13-90)	55.9 (19-84)				
Age<60	39 (100)	36(100)	163 (73.76)	27 (50.94)				
Age≥60	0	0	58 (26.24)	26 (49.06)	2.706 (1.461-5.012)	0.002	2.599 (1.084-6.232)	0.032
Sex								
Male	15 (38.46)	16 (44.44)	149 (67.42)	42 (79.25)				
Female	24 (61.54)	20 (55.56)	72 (32.58)	11 (20.75)	1.845 (0.897-3.794)	0.096		
Smoking Yes No			98 (44.34) 123 (55.66)	28 (52.83) 25 (47.17)	1.406 (0.771-2.564)	0.267		
Alcohol intake Yes No			66 (29.86) 155 (70.14)	21 (39.62) 32 (60.38)	1.541 (0.828-2.869)	0.172		
BCG vaccination								
Yes No			184 (83.26) 37 (16.74)	37 (69.81) 16 (30.19)	0.465 (0.235-0.922)	0.028	0.893 (0.342-2.330)	0.817
Past history of TB								
Yes No			26 (11.76) 195 (88.24)	4 (7.55) 49 (92.45)	0.612 (0.204-1.836)	0.381		
Body mass index(kg/m^2^)								
<18.5 ≥18.5			47 (23.74) 151(76.26)	11 (23.40) 36 (76.60)	1.019 (0.481-2.157)	0.961		
Position								
PTB			214 (96.83)	51 (96.23)				
PTB+EPTB			7 (3.17)	2 (3.77)	1.199 (0.242-5.943)	0.824		
Treatment history								
New cases			195 (88.24)	49 (92.45)				
Retreated cases			26 (11.76)	4 (7.55)	0.612 (0.204-1.836)	0.381		
Sputum smear								
Positive			104(47.06)	20 (37.74)	1.467 (0.793-2.713)	0.222		
Negative			117(52.94)	33 (62.26)				
Complication and medication								
Diabetes			52 (23.53)	16 (30.19)	1.405 (0.724-2.729)	0.315		
Hepatitis			5 (2.26)	0 (0)	0 (0.000-0.000)	0.999		
Respiratory disease			1 (0.45)	2 (3.77)	8.627 (0.767-96.994)	0.081		
Metformin use			23 (10.41)	3 (5.66)	0.517 (0.149-1.789)	0.297		
Insulin use			25 (11.31)	7 (13.21)	1.193 (0.486-2.927)	0.700		
Lymphocytes (×10^9/L) <1 ≥1			1.475±0.054 56 (25.34) 165 (74.66)	1.281±0.084 21 (39.62) 32 (60.38)	0.517 (0.276-0.969)	0.040	1.495 (0.768-2.909)	0.237

IGRA, interferon-g release assay; OR, odds ratio; PTB, pulmonary tuberculosis; EPTB, extrapulmonary tuberculosis HC, healthy control; LTBI, latent tuberculosis infection

### IFN-γ release assays

Six milliliters of blood were drawn from each participant. Once the specimen was transported to the laboratory, the peripheral blood mononuclear cells (PBMCs) of the 4 mL blood sample were separated using lymphocyte cell separation media (TBD, Tianjin, China) immediately. The PBMCs with a density of 2.5 × 10^6^ cells/mL were stimulated with an ESAT-6–CFP-10-Rv1985c fusion protein, positive control phytohemagglutinin (PHA) (P), and negative control culture medium (N) at 37°C for 16–20 h. Then cell culture supernatants were harvested for further analysis. 50 μl of supernatant was used to analyze the concentration of IFN-γ with enzyme-linked immunosorbent assay. The remaining supernatant was stored at -80°C until Luminex determinations of cytokine levels were performed. The values of MTB-specific antigen-stimulated cytokines were derived from TB antigen (T) minus negative control (N). The cut-off value was set as ≥7 pg/ml according to the manufacturer’s instruction (Deaou, Guangzhou China).

### Multiplex cytokine assay

Cell culture supernatants samples were stored frozen at −80°C prior to being subjected to Luminex xMAP technology (Luminex Austin, TX) at the same time. Cytokine concentrations in supernatants were measured using twelve test kits for cytokines (AtomLife, Nanjing, China) according to the manufacturer’s instruction. Briefly, 25 μl of antibody-linked magnetic beads suspension and 150μl of assay buffer were added to 96-well plate and washed twice. 25 μl of detection antibody and samples (tested singly) were then added, the plate sealed and shaken for 30 s at 1100 rpm then incubated for 1 h at 300 rpm. After washing, 50 μl of streptavidin-PE was added to each well and incubated for 5 min. The plate was washed again and resuspended in 150 μl of assay buffer, mixed and read immediately in the Luminex MAGPIX system with a lower bound of 50 beads per sample per cytokine. The limitation of detection for each cytokine was as follows: IL-1β [lower detection limit (LDL), 3 pg/ml and upper detection limit (UDL), 7500 pg/ml], IL-2, (LDL, 4 pg/ml and UDL, 5000 pg/ml), IL-4 (LDL, 3 pg/ml and UDL, 7500 pg/ml), IL-5 (LDL, 3 pg/ml and UDL, 7500 pg/ml), IL-6 (LDL, 2 pg/ml and UDL, 5000 pg/ml), IL-8 (LDL, 3 pg/ml and UDL, 7500 pg/ml), IL-10 (LDL, 3 pg/ml and UDL, 5000 pg/ml), IL-12p70 (LDL, 4 pg/ml and UDL, 5000 pg/ml), IL-17 (LDL, 5 pg/ml and UDL, 5000 pg/ml), IFN-γ (LDL, 5 pg/ml and UDL, 5000 pg/ml), IFN-α (LDL, 5 pg/ml and UDL, 5000 pg/ml), and TNF-α (LDL, 4 pg/ml and UDL, 5000 pg/ml).

### Statistical analysis

All statistical analyses were performed using the SPSS version 20.0 ((IBM Corp., Armonk, NY)). The continuous variables were expressed as mean (SD) or median (range), and categorical variables as count (%). We assessed the continuous variables employing the Student’s *t*-test or Wilcoxon rank-sum test depending on whether the underlying distribution of variable was normal. The univariable and multivariable logistic regression models were conducted to estimate risk factors for active TB patients with negative IGRAs. Multivariable models were built by using forward stepwise logistic regression procedures (with inclusion if *P*<0.05). The Kaplan–Meier curve was generated to compare the overall rate of bacteriological conversion over the study period. The diagnostic performance of each cytokine or cytokine combination was evaluated by a receiving operating characteristic (ROC) curve. The proportion of patients correctly diagnosed is proportional to the area under the curve (AUC). The difference was declared significant if two-sided *P* values are less than 0.05.

## Results

### Patient population

During the 16-month study period, a total of 288 laboratory-confirmed TB patients were enrolled in our study, of which 14 were excluded due to invalid IGRA results. Finally, 274 patients were included in our final analysis. Among these participants, 69.7% (191/274) patients were male and the median age was 48 years old (range from 13-90 years). The majority of patients (265/274) were only afflicted with pulmonary TB, and 89.1% (244/274) were treatment-naïve cases.

### Risk factors associated with TB patients with negative IGRA results

Of the 274 enrolled patients, 221 (80.7%) were IGRA positive and 53 (19.3%) were IGRA negative. We summarized characteristics of IGRA-negative patients compared with IGRA-positive patients. The distribution of IGRA negative patients also differe among age groups. Using patients < 60 years of age as a control group, we found that elder persons exhibited higher odds of yielding negative IGRA results cOR 2.706, 95% CI 1.461-5.012. In the multivariable logistic regression model, only elder persons were identified as an independent risk factor for negative IGRA results aOR 2.599, 95% CI 1.084-6.232. Although the patients with negative IGRA results seemed to have a lower count of blood lymphocytes than patients with positive IGRA results (1.281 ± 0.084×10^9^/L for IGRA-negative group versus 1.475 ± 0.054×10^9^/L for IGRA-positive group), statistical analysis revealed that this difference was not significant.

### Cytokine profiles of TB antigen-specific T Cells

**Figure 1:**
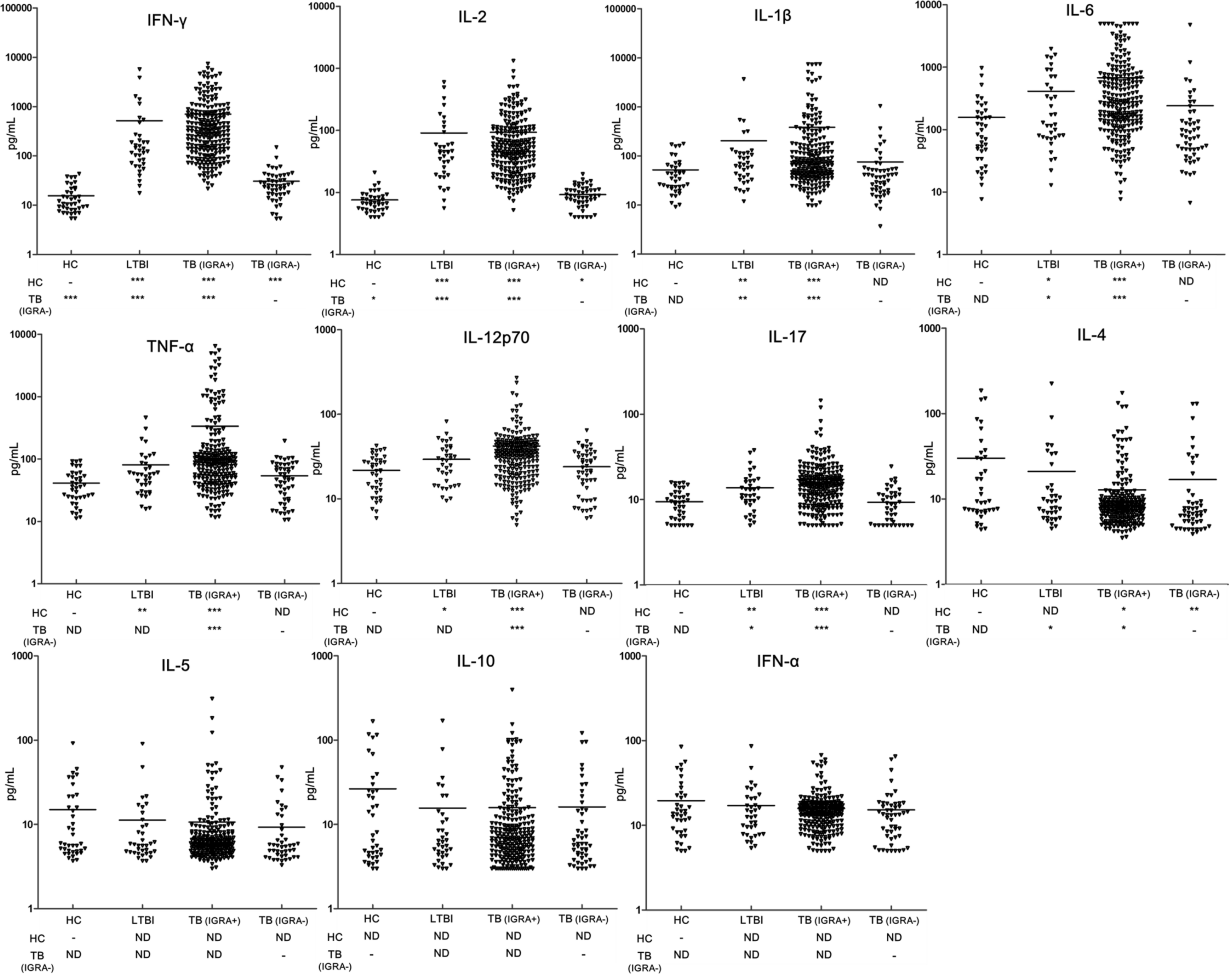
Comparison of the concentration of multiple cytokines induced by MTB-specific antigens between groups. Levels of cytokines were detected by using Luminex xMAP technology in supernatant of PBMCs from patients with IGRA-positive TB group (TB (IGRA+), n=221), IGRA-negative TB group (TB (IGRA-), n=50), individuals with latent TB infection (LTBI, n=36), and healthy controls (HC, n=39). The data are represented as scatter plots with each triangular representing a single individual. Statistical differences were analyzed using Wilcoxon rank-sum test; * *P<*0.05, ** *P*<0.01 and *** *P*<0.001.

In order to determine the potential mechanism for impaired release of IFN-γ by TB-specific T cells, we recruited 39 healthy control and 36 individuals with LTBI in our study. **Figure 1** illustrates the comparative analysis of cytokine profiles of TB antigen-specific T cells across four populations. Overall, IGRA-negative group exhibited comparable cytokine profiles to healthy control group after stimulation with TB antigens, and only slightly higher IFN-γ and IL-2 and lower IL-4 levels were noted in IGRA-negative group. In contrast, the individuals of LTBI and TB patients with positive IGRA results had significantly higher levels of multiple proinflammatory cytokines, including TNF-α, IL-12p70, and IL-6. Moreover, compared with patients with positive IGRA results, a remarkedly increased level of anti-inflammatory IL-4 was observed in patients with negative IGRA results (*P*<0.05).

### ROC analyses

**Figure 2:**
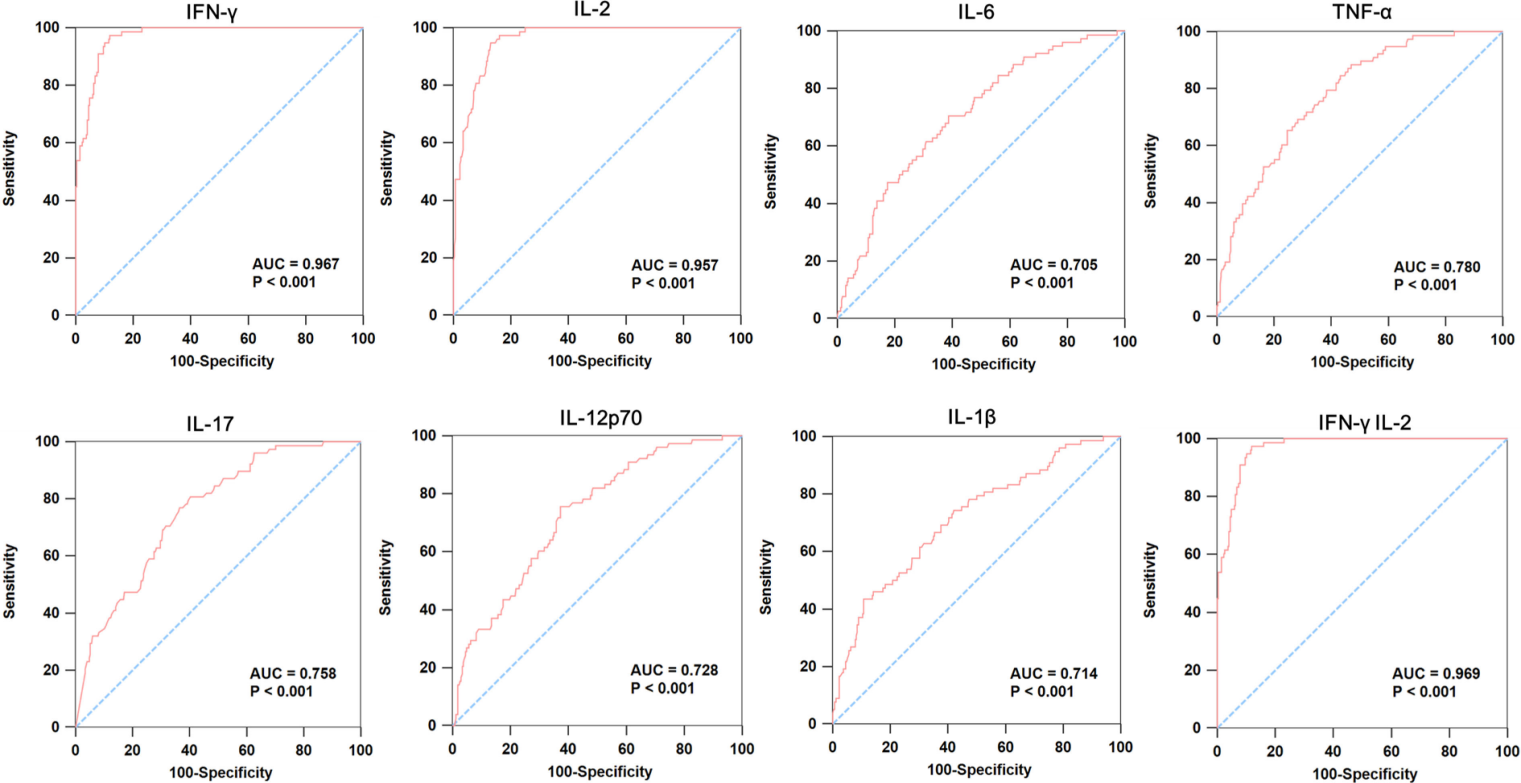
ROC curve analysis of cytokines in individuals with latent TB infection and IGRA-positive TB patients compared to healthy control and IGRA-negative TB patients. We combined LTBI and IGRA-positive TB patients as Group I, and healthy control and IGRA-negative TB patients as Group II. Then, we did the ROC curve to analysis the expression of IFN-γ, IL-2, IL-6, TNF-α, IL-17, IL-12p70, IL-1β and IFN-γ&IL-2 in these two groups.

Given the significant difference in cytokine levels of IGRA-negative group compared with LTBI and IGRA-positive group but with high similarity with healthy control, we proposed a stepwise algorithm for the diagnosis of IGRA-negative group. First, LTBI and IGRA-positive TB patients were combined as Group I, and healthy control and IGRA-negative TB patients were combined as Group II. As expected, ROC analyses comparing Group I and Group II samples demonstrated that IFN-γ and IL-2 had very good accuracy for distinguishing these two groups, yielding the greatest AUC of 0.967 and 0.957, respectively. The combination of IFN-γ and IL-2 could provide the most excellent accuracy for discrimination of Group I and II, which had an AUC of 0.969 (**Figure 2**). However, none of them provided acceptable accuracy to discrimination between LTBI and IGRA-positive groups (see **Supplementary Figure S1**).

**Figure 3:**
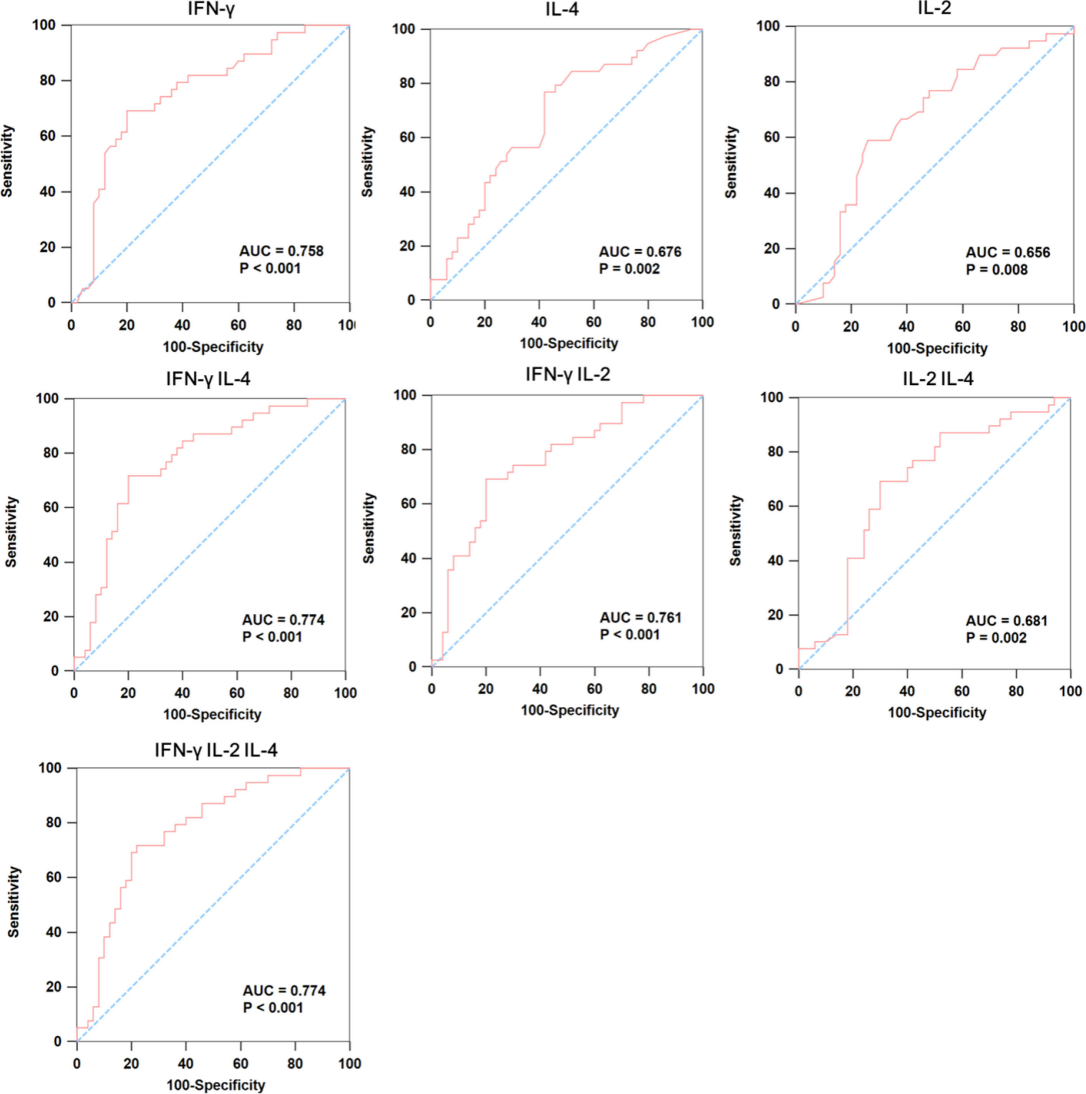
The ROC curve analysis of cytokines between IGRA-negative TB patients and healthy controls. We used ROC curve analysis to detect whether the combination of multiple cytokines could be used as promising markers for differentiating IGRA-negative group (n=50) from healthy controls (n=39), where the AUC was calculated to evaluate the severity and prognostic accuracy of each marker.

We further analyzed whether TB-specific cytokines could be used as promising markers for differentiating between healthy control and IGRA-negative group. As shown in **Figure 3**, IFN-γ, IL-4, and IL-2 exhibited moderate discriminatory power with AUC values higher than 0.65 in discriminating healthy control from TB patients with negative IGRA results. Using combinations of multiple cytokines, we found that the combination of IFN-γ, and IL-4 exhibited the greatest AUC of 0.774, which was comparable with that derived from IFN-γ, IL-4, and IL-2 combination (AUC=0.774).

**Figure 4:**
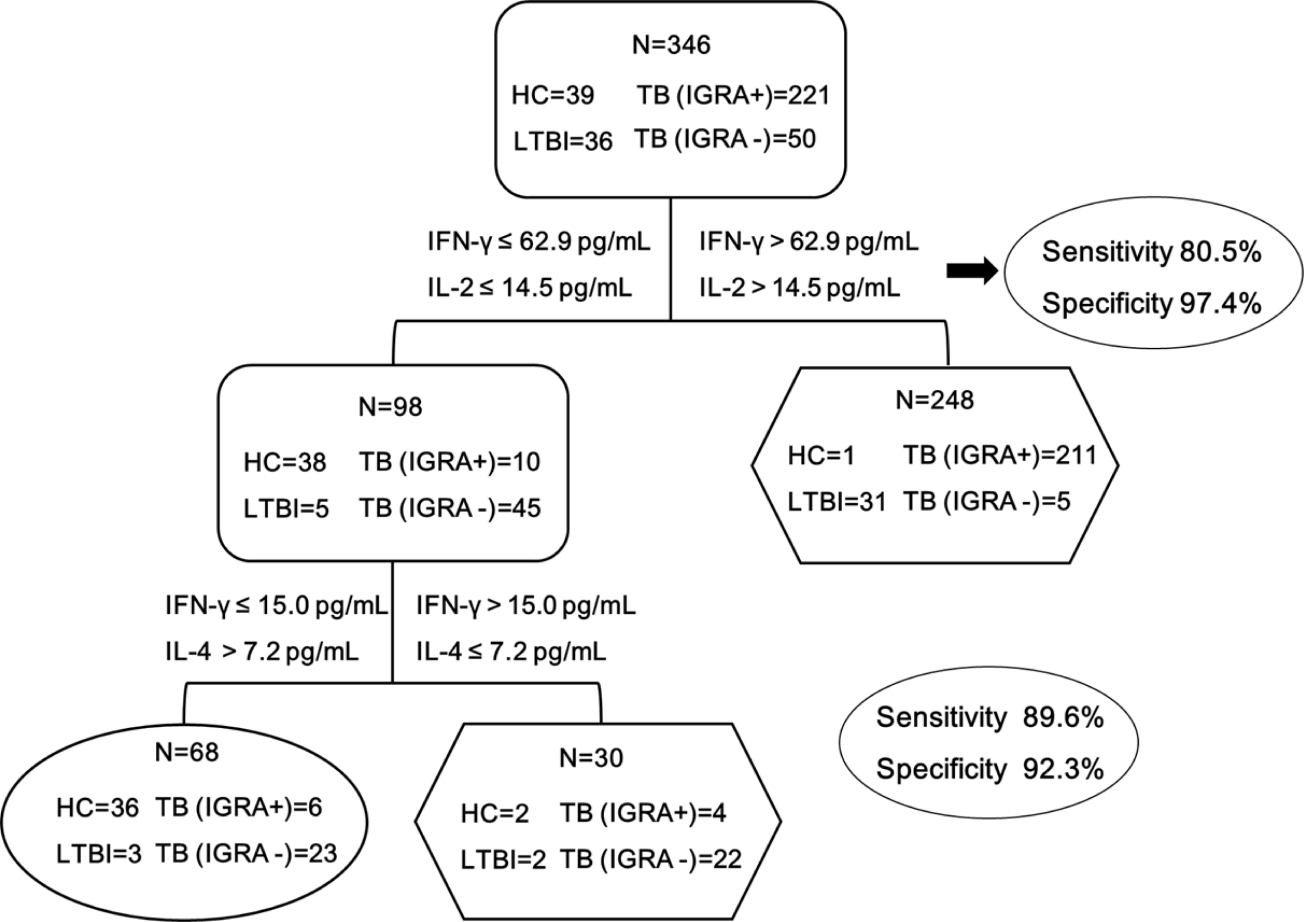
The combination of IFN-γ&IL-2 and IFN-γ&IL-4 provides the best discrimination between HC and MTB infection groups. MTB infection group was defined as TB (IGRA+) patients, TB (IGRA-) patients, LTBI group, while HC group was defined as the control for analyzing the diagnostic performance of the biomarkers. Diagnostic strategy and optimum cut-offs of IFN-γ、IL-2 and IL-4 were decided by ROC analysis. The sensitivity and specificity of the IFN-γ&IL-2 and IFN-γ&IL-4 panel were 89.6% and 92.3% respectively. TB (IGRA+) group, n=221; TB (IGRA-) group, n=50; LTBI group, n=36; HC group, n=39. Oval and hexagon showed the number finally determined as HC and MTB infection, respectively.

We further assessed the performance of the stepwise algorithm for the diagnosis of MTB-infected patients. As shown in **Figure 4**, the conventional IFN-γ&IL-2 based assay could correctly identify 247 out of 307 MTB-infected patients, demonstrating a sensitivity of 80.5% (95% CI: 76.4-85.2). In addition, the use of the stepwise algorithm allowed us to additionally detect IFN-γ&IL-4 MTB-infected patients. Correspondingly, the sensitivity was increased from 80.5% to 89.6% (95% CI: 86.5-93.3), and statistical analysis revealed that this difference was significant (*P*=0.001).

### Culture conversion intervals based on IGRA results

**Figure 5:**
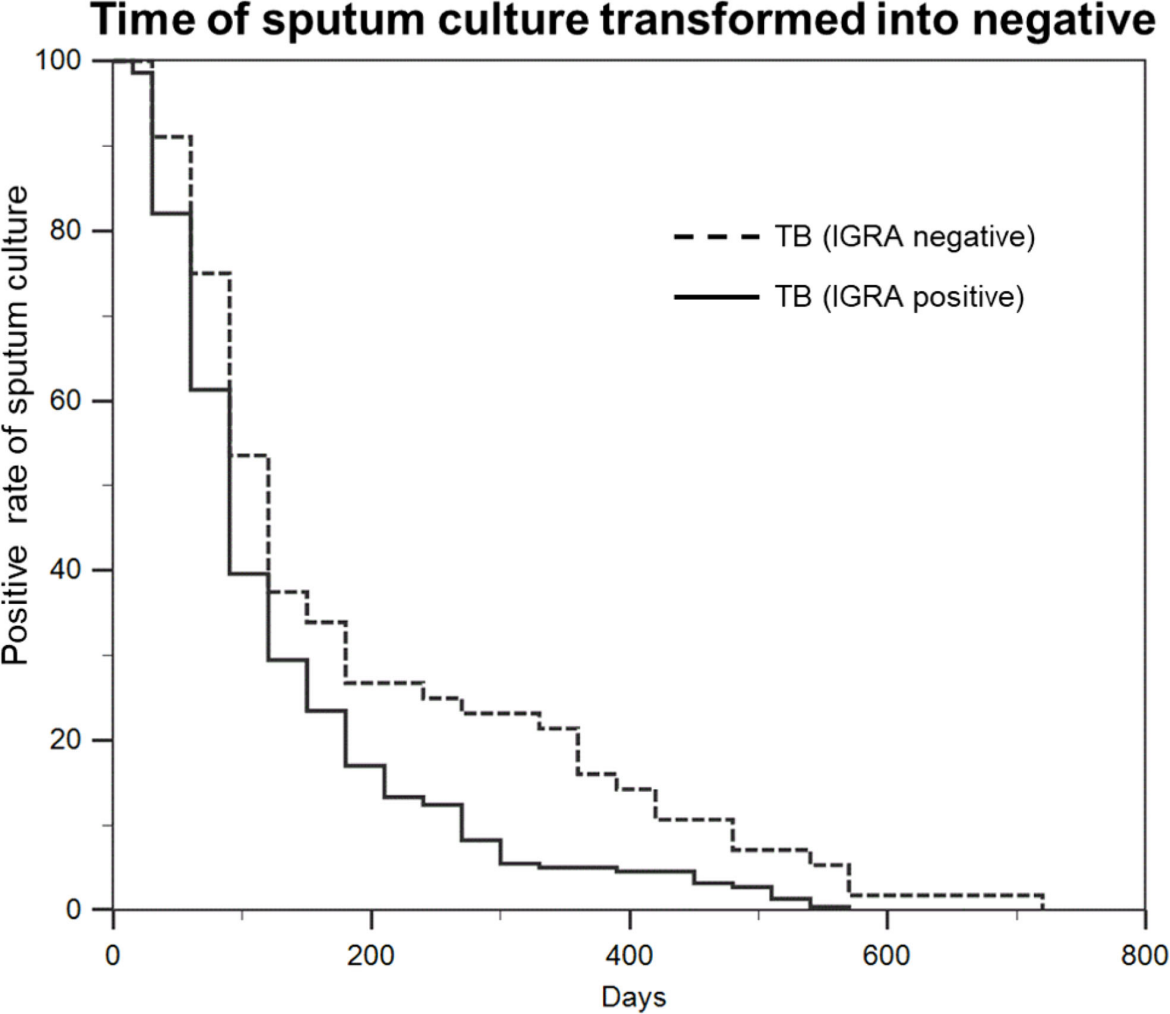
Patients with negative IGRA have a conversion to culture-negative status longer than those with positive IGRA. Statistical analysis of the time of sputum culture transformed into negative between TB (IGRA positive) group and TB (IGRA negative) group.

Given the decreased production of proinflammatory cytokines of TB-specific T cells in patients with IGRA-negative results, an interesting question was raised about whether the impaired T cell response had a negative impact on the clinical outcomes of these patients. Out of 274 active TB patients in our analysis, a total of 273 patients had detailed follow-up data. As summarized in **Figure 5**, patients with negative IGRA results had a conversion to culture-negative status longer than those with positive IGRA results by using mycobacterial culture with MGIT (P=0.019). We compared the time of sputum culture transformed into negative between patients with negative IGRA group and patients with positive IGRA group at completion of 90 days of treatment. 27(50.9%) of 53 individuals remained culture positive in patients with negative IGRA group, and 89(40.5%) of 220 individuals remained culture positive in patients with positive IGRA group. The average time for sputum culture transformed into negative in patients with negative IGRA group was 5.85 ± 0.77 months, and 4.30 ± 0.26 months in patients with positive IGRA group.

## Discussion

The false negative IGRA results remain an unsolved diagnostic dilemma requiring a high index of clinical suspicion. The sensitivity of IGRAs exhibited great diversity across studies, ranging from 60% (17) to 99% (18). In a published systematic review, the pooled sensitivity for the diagnosis of active TB was 81% for IGRA assay (11). In our analysis, the overall sensitivity of IGRA (80.7%) was similar to the pooled sensitivity of previous meta-analysis. However, approximately one-fifth of active TB patients were missed by IGRA results, which limits the clinical usage of IGRA assays. On one hand, the diagnostic sensitivity of these tests is not sufficient to be used as an individual method for detecting active TB patients. The negative results should be interpreted in combination with other laboratory assays and clinical findings. On the other hand, although the IGRAs provide promising accuracy for identifying MTB infections, we should acknowledge that the negative predictive value is not sufficiently high, especially for patients at high risk for development of active TB. For patients receiving immunosuppressive agents (19), cautious notification and continual monitoring are necessary to timely identify the emergence of tubercle bacilli regardless of IGRA results.

Several risk factors for false negative IGRA results have been reported, including advanced age and low peripheral lymphocyte counts (13, 15). In line with previous reports (13), older age was the only independent factor associated with a negative IGRA test in our active TB cohort. Aging has negative regulatory effects on the immune system, especially adaptive immune responses (20). Importantly, the more profound defects are found when aged naïve CD4 T cells that transition to memory cells reencounter antigen (21, 22). Previous experimental studies have demonstrated that these memory cells originated from aged naïve CD4 T cells secrete an impaired pattern of cytokines and expanded little after restimulation (21). A published study by Channappanavar and coresearchers revealed that the predominance of inhibitory receptor expressing CD4 T cells is a plausible mechanism for age-related defects in elderly individuals (23). In contrast to previous investigations (15), low peripheral lymphocyte counts could not be identified as a risk factor for false negative IGRA results in our cohort. This heterogeneity across studies may be explained that the IGRA method used herein requires a specific number of PBMCs rather than the methods that use whole blood without any standardizations of the number of mononuclear cells.

Another interesting finding of our report was the elevated level of IL-4 and the decreased levels of IFN-γ, IL-2, IL-6, IL-1β, and IL-12 in IGRA-negative TB relative to IGRA-positive TB group, demonstrating a shift in the Th1/Th2 between two groups. It is well known that T cell-mediated immunity to MTB infection is mediated by Th1 CD4 cells secreting IFN-γ, as opposed to Th2 CD4 cells secreting IL-4 and IL-10 (24). In a previous clinical trial, increased production of IL-4 by T cells from TB patients was related to the presence of pulmonary cavities (25), indicating the role of IL-4 in antagonizing host defense and resulting in tissue damage. It may be partly explained that the high expression level of inhibitory receptors in aged Th1 cells could inhibit T cell receptor clustering (26), thereby regulating the Th1/Th2 cytokine production. In view of the predominant role of Th1 cytokines against tubercle bacilli, the down-regulation of the Th1 response would increase host susceptibility to MTB infections and consequently prevent efficient clearance of MTB. Consistent with our hypothesis, the patients with negative IGRAs underwent a delayed culture conversion compared with those with positive IGRAs, suggesting that the impaired Th1 response to MTB stimulation has a negative effect on clinical outcomes of active TB patients. Further studies are warranted to determine what options would reverse impaired memory T cells, and elucidate its correlation with clinical outcomes for this population.

Based on the cytokine profiles of TB antigen-specific T cells among different groups, we put forward a stepwise algorithm for the identification of MTB infections. Specifically, the combination of IFN-γ and IL-4 could moderately differentiate patients with negative IGRA results from healthy controls. Although its specificity was 92.3% at a sensitivity of 89.6%, we believe that it would provide additional benefits for immunosuppressive individuals to accurately diagnose MTB infections. In comparison with conventional IGRAs, the combination of IFN-γ, IL-2, and IL-4 achieves increased diagnostic sensitivity for TB infections.

We also acknowledged several obvious limitations to the present study. First, we only included active patients having negative IGRAs rather than LTBI group given the lack of uniform diagnostic criteria. Hence, further study is needed to evaluate the accuracy of our diagnostic algorithm. Second, there is evidence that false negative IGRA results are noted more frequently in younger children and HIV-positive participants. However, because there were no children aged < 13 years and HIV-positive patients enrolled in this study, we could not determine whether they were risk factors for false negative IGRA results. Third, the growing research on comparative studies of cytokine levels in cultures of human whole blood and PBMCs demonstrated that whole blood assays correlated well with PBMC cultures (27–29); inversely, poor correlation was noted in the IFN-γ levels in the two culture systems by Silberer and colleagues (30). In this study, we only detected the cytokine levels using PBMC cultures rather than whole blood, which may limit tis application. Fourth, the diagnosis of TB remains clinically challenging due to lack of point-of-care testing. Unfortunately, because the detection of TB-specific cytokines requires several hours of antigenic stimulation for commitment to cell activation, this diagnostic assay does not meet the criteria for point-of-care testing. Further study is required to identify novel biomarkers that could detect MTB infections at the point-of-care. Finally, although we also aimed to distinguish between active TB patients and LTBI, the cytokine profiles of TB-specific T cells exhibited a high degree of similarity between active TB and LTBI groups, which hampered the achievement of our primary objective. Nonetheless, our preliminary data indicated that the bacterial burden was not correlated with TB-specific cytokine response, thus challenging the assumption of whether IGRA could be applied as a surrogate marker for treatment response of active patients (31, 32).

In conclusion, our data demonstrate that approximately one-fifth of active TB patients are missed by IGRA results. Older age is the only independent factor associated with a negative IGRA test in our active TB cohort. In addition, the elevated level of IL-4 and the decreased levels of IFN-γ, IL-2, IL-6, IL-1β, and IL-12 in IGRA negative TB relative to IGRA positive TB group, demonstrating a shift in the Th1/Th2 between two groups. The patients with negative IGRAs undergo a delayed culture conversion compared with those with positive IGRAs. Additionally, a stepwise algorithm outperforms IGRA assays to accurately identify MTB infections by the combination IFN-γ, IL-2, and IL-4. Further study is needed to evaluate the accuracy of our diagnostic algorithm in the LTBI population.

## Data availability statement

The original contributions presented in the study are included in the article/**Supplementary Material**. Further inquiries can be directed to the corresponding authors.

## Ethics statement

The studies involving human participants were reviewed and approved by Ethics committee of Beijing Chest Hospital, Capital Medical University (approval number: YJS-2019-016). Written informed consent to participate in this study was provided by the participants’ legal guardian/next of kin.

## Author contributions

QL, MG, and YP participated in the study design. QL, WR, JY, HG, YS, WW, JP, MG and YP collected the data. QL, WR, JP, MG, and YP analyzed and interpreted data. QL, MG, and YP wrote the first draft of this report. All authors gave input to the final version. JP, MG, and YP had the final responsibility for the decision to submit the study for publication. All authors contributed to the article and approved the submitted version.

## Funding

This work was supported by the Beijing Hospitals Authority Ascent Plan (DFL20191601), the Beijing Hospitals Authority Clinical Medicine Development of Special Funding (ZYLX202122), Beijing Key Clinical Specialty Project (20201214) and Tongzhou lianggao talents project (No. YHLJ202005). The funders had no role in study design, data collection, analysis, interpretation or writing of the report.

## Acknowledgments

We would like to thank all the staffs participating this study from Beijing Chest Hospital.

## Conflict of interest

The authors declare that the research was conducted in the absence of any commercial or financial relationships that could be construed as a potential conflict of interest.

## Publisher’s note

All claims expressed in this article are solely those of the authors and do not necessarily represent those of their affiliated organizations, or those of the publisher, the editors and the reviewers. Any product that may be evaluated in this article, or claim that may be made by its manufacturer, is not guaranteed or endorsed by the publisher.
