# Supplementary figures and images for: Significant difference in Th1/Th2 paradigm induced by tuberculosis-specific antigens between IGRA-positive and IGRA-negative patients
Source: Front Immunol. 2022 Aug 31;13:904308. doi: 10.3389/fimmu.2022.904308 (PMC9471257; doi:10.3389/fimmu.2022.904308)

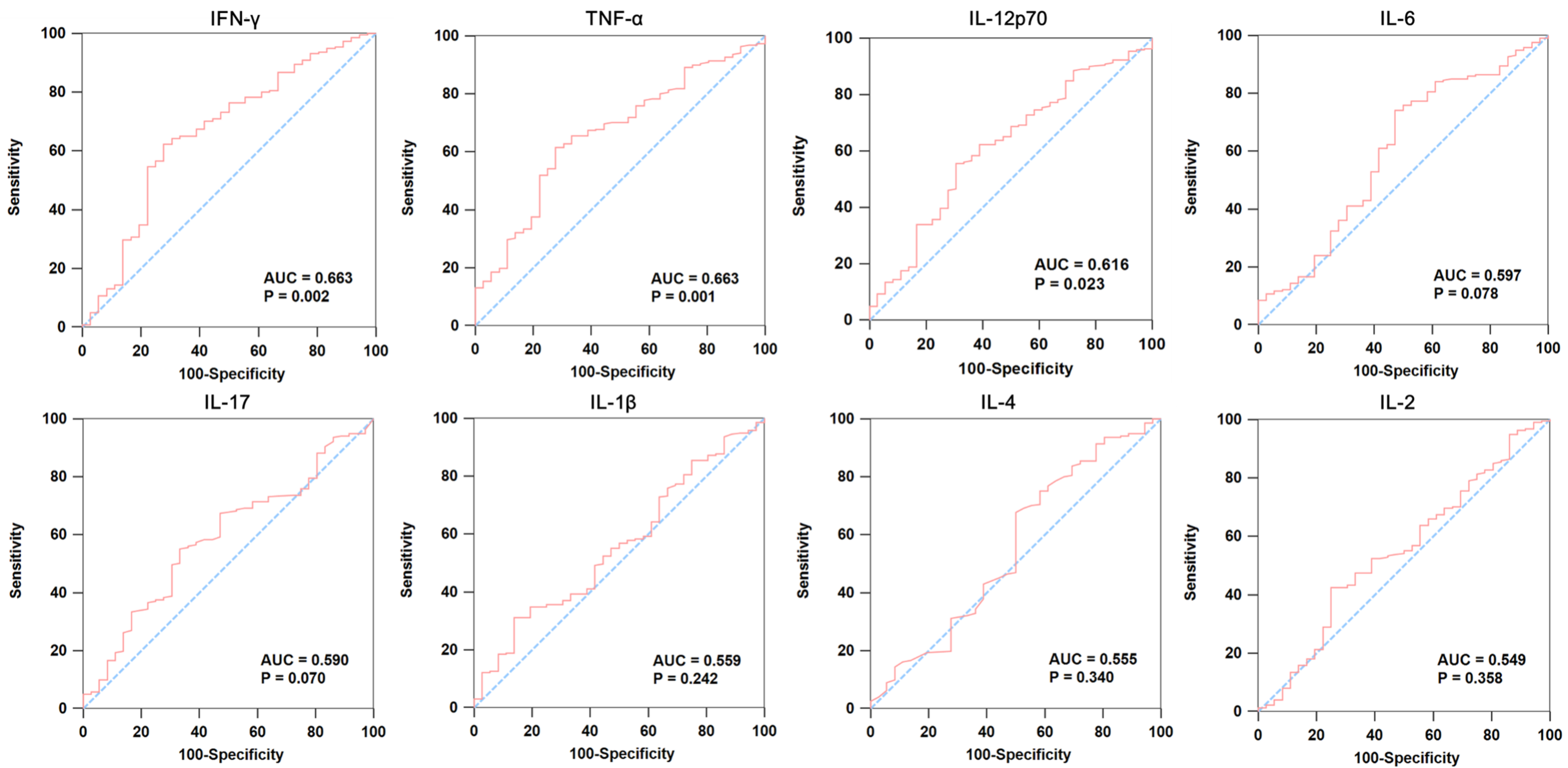

Supplement: Supplementary file 1 [file Image_1.tif]
